# The Role of CDK4 in the Pathogenesis of Pancreatic Cancer

**DOI:** 10.3390/healthcare9111478

**Published:** 2021-10-30

**Authors:** Emily Jiggens, Maria Mortoglou, Guy H. Grant, Pinar Uysal-Onganer

**Affiliations:** 1 Cancer Research Group, School of Life Sciences, University of Westminster, London W1W 6UW, UK; w1712684@my.westminster.ac.uk (E.J.); w1754188@my.westminster.ac.uk (M.M.); 2 Department of Life Sciences, University of Bedfordshire, Park Square, Luton LU1 3JU, UK; guy.grant@beds.ac.uk

**Keywords:** pancreatic cancer, CDK4, CDK4/6 inhibitor, epidemiology

## Abstract

Pancreatic cancer (PC) continues to have the lowest overall survival and the lack of effective early diagnosis. Cyclin-dependent kinase 4 (CDK4) plays a fundamental role in the orderly progression of the cell cycle, binding to cyclin D to promote the progression through the G1/2 transition. The inhibition of CDK4/6 has therefore gained substantial interest in the hope of new and effective therapeutics in multiple cancers, such as advanced metastatic breast cancer. While the use of these agents is encouraging, their potential is yet to be fully explored. In this study we used the GLOBOCAN database to understand the most recent epidemiology of PC, Human Protein Atlas and KEGG to highlight the role, prevalence, and significance on patient survival of CDK4 in PC. We found that CDK4 cannot be used as prognostic in PC and no significant differences were observed between CDK4 expression and the patient’s clinical status, though larger studies, especially concerning CDK4 protein expressions, are required for a more thorough understanding. The use of CDK4/6 inhibitors in PC is still in clinical trials. However, due to only modest improvements observed in the use of single-agent therapies, efforts have focused on combinatorial approaches.

## 1. Introduction

Pancreatic Cancer (PC) is a highly aggressive malignancy, which continues to have the lowest overall 1- and 5-year patient survival rates amongst all cancer sites in the United Kingdom, at 25% and 7%, respectively [1]. While surgical resection remains the only curative treatment available, 80–85% of tumours are diagnosed at late and unresectable stages, with evidence of locally advanced or metastatic disease. For the small proportion of patients that do qualify for surgical care, survival rates are modest - despite numerous benefits observed when studying the early detection of PC [2] - and patients frequently suffer from local recurrence, metachronous metastasis and high tumour chemoresistance following resection, seen in up to 80% of cases [3]. Despite advances in diagnostic technology, the high frequency of late-stage diagnoses, and the associated poor prognosis, is largely attributable to the non-specific early-stage symptoms, the lack of specific and sensitive biomarkers, and the retroperitoneal position of the pancreas that restricts the accessibility of routine checks [4]. 

Pancreatic ductal adenocarcinoma (PDAC) is an invasive pancreatic epithelial neoplasm with glandular differentiation and is the most prevalent histological subtype of PC, accounting for approximately 85% of pancreatic tumours; the terms PC and PDAC are often used synonymously. In recent studies, several subtypes of PDAC have been defined following gene expression analysis. The implications they hold for future precision medicine and targeted patient clinical care is promising: results show that the presence of stromal infiltration improves survival in patients with desmoplastic or stroma-activated subtypes, whereas basal-like tumours have an unfavourable prognosis and a poor median overall survival (OS) rate of 10.3 months. However, these lack consensus and a universally agreed classification system is required for implementation into routine clinical practice [5]. 

Conversely, pancreatic neuroendocrine tumours (PanNET) occur in the endocrine tissue of the pancreas and account for less than 5% of PC cases [6]. They are classified according to the Ki-67 index, and the 5-year patient survival rates are substantially greater than those seen in PDAC, ranging from 29% for poorly differentiated, and 60–100% for well-differentiated PanNETs [7]. 

As most PC patients present at an already advanced stage, major efforts have focused on determining the biological hallmarks of how normal epithelium is transformed into invasive carcinomas, in the hope of highlighting targets for new biomarkers and therapeutics. In recent years, due to the success of extensive genomic and histological studies, non-invasive precursor lesions that precede the development of PC have been defined and categorised based on their biological and clinical behaviours; those most commonly studied are pancreatic intraepithelial neoplasia (PanIN) and intraductal papillary mucinous neoplasms (IPMN) [8]. 

Precursor lesions advance, and pancreatic cells acquire malignant behaviour in a stepwise progression following the accumulation of genetic alterations guided by four driver mutations. During early events, *KRAS*, observed in 90% of PC tumours, is mutated at several hotspots. The *KRAS* gene protein product is responsible for cell differentiation and proliferation by binding to GTP. Oncogenic mutations of *KRAS* therefore prevent the hydrolysis of GTP resulting in the permanent activation of RAS effector pathways, including MAPK and P13K/AKT, causing uncontrolled cell proliferation [9]. This is followed by the inactivation of several tumour suppressor genes, including *CDK2NA*, *TP53* and *SMAD4* [10]; though the order in which these occur has been hugely debated. 

The histological progression of PanINs, the most common and important precursor of PC, follows this multi-step model closely. However, the accumulation of genetic alterations confers a higher grade of differentiation [11]. Low grade PanINs, which are either flat (PanIN-1A) or papillary (PanIN-1B) epithelial lesions with basally located nuclei, usually harbour *KRAS* mutations. Intermediate grade (PanIN-2) lesions have been associated with the inactivation of *CDKN2A*/p16^INK4a^ gene, which usually encodes the tumour suppressor protein p16^INK4a^ gene that binds to cyclin-dependent kinase (CDK) 4/6 to arrest the cell cycle in G1 phase. This consequential loss of p16^INK4a^ results in the loss of apoptosis and control of the cell cycle causing unregulated cell proliferation. The final steps in the progression of high-grade (PanIN-3) lesions to invasive carcinoma are associated with mutations in *TP53*, *DPC4* and *BRCA2* [12]. 

CDKs are serine/threonine kinases, which are activated by specific cyclins to form CDK/cyclin-complexes that play a fundamental role in the orderly progression of the cell. There are 21 known CDKs that are classified and grouped according to their role in cell cycle regulation, regulation of transcription, and those that have unique tissue-specific functions [13]. 

As part of the G1 phase of the cell cycle, the synthesis of cyclin D is stimulated, which forms a complex with CDKs 4 and 6 (classified based on their role in cell cycle regulation) to promote cell cycle entry and the progression through the G1 and G1/S transition [14]. Since the aforementioned loss of p16^INK4a^ is a common feature of *KRAS*-driven PC, the inhibition of CDK4/6 has gained substantial interest as a potential therapeutic reatment for p16^INK4a^-deficient tumours. CDK4/6 inhibitors (CDK4/6i), such as Palbociclib, Ribociclib and Abemaciclib, have recently gained FDA-approval and shown success in the treatment of advanced metastatic hormone receptor-positive (HR+)/HER2-negative breast cancer [15]. However, previous studies have shown that the use of single-agent therapies with CDK4/6i offer only modest improvements in most cancer types, therefore clinical trials are testing CDK4/6i combined with chemotherapy across a number of malignancies, including PC [16]. While the use of these agents is encouraging, their potential is yet to be fully explored, in part due to their novelty. 

There is a clear, and as yet unmet, need for adequate and effective chemotherapeutic regimens for PC, as few patients qualify for resection at the time of their diagnosis and are instead treated with palliative chemotherapy, which offers unfavourable results. Therefore, this study aims to provide an up-to-date review of the role, prevalence and significance on patient survival of CDK4 in PC, in relation to understanding the potential efficacy of CDK4/6 inhibitors as a combined treatment option with chemotherapy for PC patients by evaluating the current epidemiology of PC and the expression levels of CDK4 in PC tissues, obtained from open-access online databases. 

## 2. Materials and Methods

This in silico study analyses data already available from online databases, as described in detail below. 

### 2.1. Pancreatic Cancer Epidemiology

The global incidence and mortality estimates of PC in 2020 were obtained from the GLOBOCAN database (International agency for Research on Cancer, Lyon, France) [17], available for 184 countries. To assess geographic variations and the overall global burden of PC, the data were extracted for the pancreatic cancer type (C25) for each sex by continent and Human Development Index (HDI)—where HDI is the composite index of life expectancy at birth, education period, and gross national income per capita [18]. Since age has a strong influence on the risk of cancer, results include the age-standardised rates (ASR) per 100,000, defined as the summary rate that would have been observed if the population had a standard age structure [19]. The cumulative risks, where the risk is calculated as the number of newborn children (out of 100) expected to develop/die from PC over a lifetime, were also reported. Additionally, data were obtained from GLOBOCAN for the number of PC cases and deaths for both males and females between the years 1995 and 2012 to assess any trends over time; data were unavailable for years outside of this range [20]. 

The methods used by GLOBOCAN to collate the 2020 estimates were modelled based on incidence-mortality ratios and the approximation from neighbouring countries, similar to methods used in their estimates in 2012 and 2018. The exact sources and methods used are described online at the Global Cancer Observatory (GCO) (gco.iarc.fr, accessed on 1 June 2021) [21]. The consequent data were displayed and analysed using Microsoft^®^ Excel 2021 version 16.50 (Microsoft Corporation, Washington, DC, USA). 

### 2.2. CDK4 Expression in Pancreatic Cancer Tissues

Data were obtained from the Human Pathology Atlas as part of the Human Protein Atlas (HPA) available from http://www.proteinatlas.org (accesssed on 20 May 2021) [22]. HPA allows for the exploration into the prognostic role of protein-coding genes for each cancer type by the use of transcriptomics and antibody-based profiling. On the online, open-access program, “CDK4” and “pancreatic cancer” were selected, which provided the data used to carry out this study [23]. 

#### 2.2.1. FPKM RNA-Seq

The RNA-sequence data available from the HPA was obtained from The Cancer Genome Atlas (TCGA) project of Genomic Data Commons (GDC) using the Ensembl gene ID (CDK4; ENSG00000135446) available from TCGA. The FPKMs (number of Fragments Per Kilobase of exon per Million reads) for the gene were used for the quantification of gene expression with a detection threshold of 1 FPKM. Clinical metadata (age, stage, ethnicity, time, sex) was also available for each patient. 

For this study, data were collected for 173 PDAC patients (out of 176 available; 3 were excluded as data were missing specifying the patient’s cancer stage). SPSS Statistics for Macintosh, version 27.0 (IBM, New York, NY, USA) was used to run statistical analysis, including Kaplan-Meier survival analysis, Kruskal–Wallis, Mann–Whitney U test of difference and Spearman’s rank correlation coefficient. 

#### 2.2.2. Antibody Profiling

Histological sections from 12 PDAC patients, stained by immunohistochemistry with two separate antibodies specific for CDK4, were available on the HPA database [22]. The two antibodies used to assess CDK4 protein expression on the PDAC cells are: (i) mouse monoclonal antibody CAB013116 (*n* = 11), manufactured by ThermoFisher Scientific (Waltam, MA, USA) and (ii) mouse monoclonal antibody CAB069405 (*n* = 12), manufactured by AbFrontier Co., Ltd. (Seoul, South Korea). The antibodies were labelled with DAB (3,3′-diaminobenzidine), resulting in a brown staining that indicates when the antibody has bound to its antigen. All images of tissues stained by the immunohistochemical protocol [24] were then manually annotated by a specialist and verified by a second specialist, using fixed guidelines for classification of immunohistochemical results. The annotation parameters included are: (i) staining intensity (negative, weak, moderate, or strong), (ii) fraction of cell (<25%, 25–75% or >75%) and (iii) subcellular location (nuclear and/or cytoplasmic/membranous). 

The histological images (11 for CAB013116; 12 for CAB069405) were downloaded from the HPA and the corresponding annotations and patient information data (sex, age) were exported to SPSS Statistics for Macintosh, version 27.0 (IBM, New York, NY, USA) for statistical analysis. 

### 2.3. The Normal and Pathological Role of CDK4

To investigate and visualise the biological function of CDK4, the Kyoto Encyclopedia of Genes and Genomes (KEGG) PATHWAY database (Kyoto, Japan) [25] was searched. The search results were then recorded, and examples of the different pathways/diseases CDK4 and the accompanying maps are provided. 

## 3. Results

### 3.1. Incidence, Mortality and Comparison by HDI of PC in 2020

Globally, an estimated 495,773 new cases of PC and 466,003 associated deaths were reported in 2020 (Table 1). The male to female ratio is 1.13:1 for both global incidence and mortality rates. The highest incidence rates, when comparing ASR per 100,000, were seen in Northern America (ASR, 8), Europe (ASR, 7.8) and Oceania (ASR, 6.6) and the lowest in Africa (ASR, 2.3). The risk of developing PC was shown to be greatest in Northern America (2.7%) and European males (2.67%) and lowest in African females (0.58%) and males (0.79%) (Table 1). The highest mortality rates were also seen in Europe (ASR, 7.2) and Northern America (ASR, 6.5) and the lowest in Africa (ASR, 2.3). 

**Figure 1:**
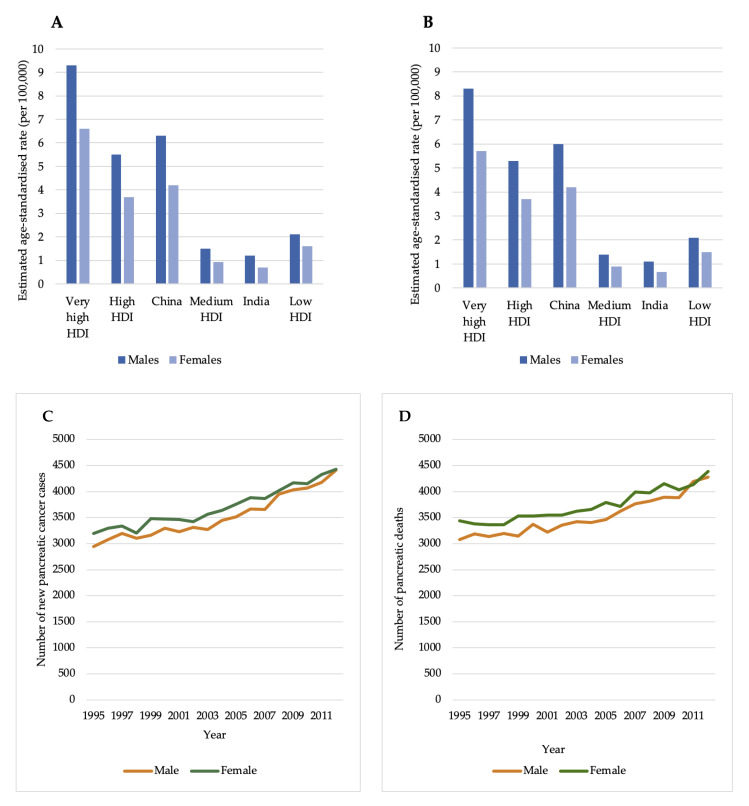
Estimated age-standardised (ASR) incidence (**A**) and mortality (**B**) rates for pancreatic cancer in 2020 to reflect international levels of education, social and economic development, using the four-tier Human Development Index (HDI) system, and trends in the estimated number of cases (**C**) and deaths (**D**) of pancreatic cancer in the United Kingdom between the years 1995 and 2012 in males and females of all ages (0–85+) Adapted from: GLOBOCAN, 2020 [17,20].

The extent to which the development of, or death from, pancreatic cancer reflects international levels of social and economic development is shown in Figure 1. Countries with very high HDI had the greatest incidence of PC for both males (ASR, 9.3) and females (ASR, 6.6), with the lowest incidences found in countries with medium HDI (ASR, 1.5 and 0.92 for males and females respectively) (Figure 1A). A similar trend was seen in mortality rates, with countries with a very high HDI having the greatest mortality rates, and countries with a medium HDI experiencing the lowest (Figure 1B). Between the years 1995 and 2012, there has been an increase in both the number of new cases and deaths of PC for males and females (Figure 1C,D). The average annual increase for incidence rates is 2% in both males and females (Supplementary Figure S1) and the average annual increase for mortality rates is 2% and 1% for males and females, respectively (Supplementary Figure S2). 

### 3.2. FPKM RNA-Seq of CDK4

#### 3.2.1. Descriptive Statistics

**Figure 2:**
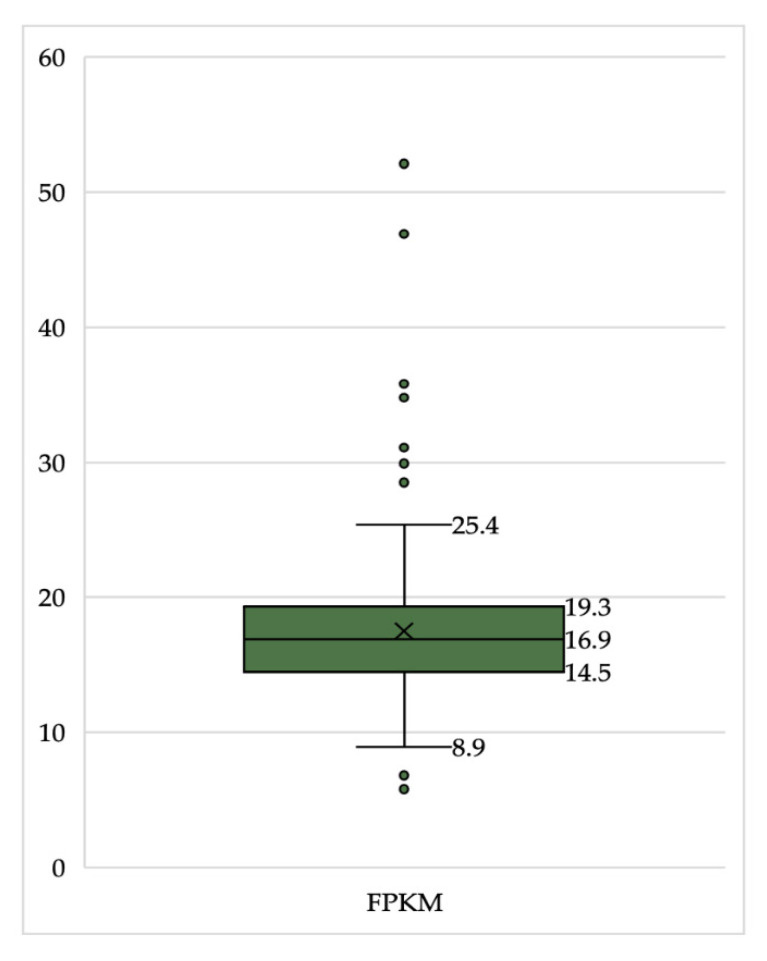
A box plot demonstrating the expression of FPKM (quantification of CDK4) in 173 pancreatic ductal adenocarcinoma patients. Interquartile range, median and upper/lower bounds shown.

**Figure 3:**
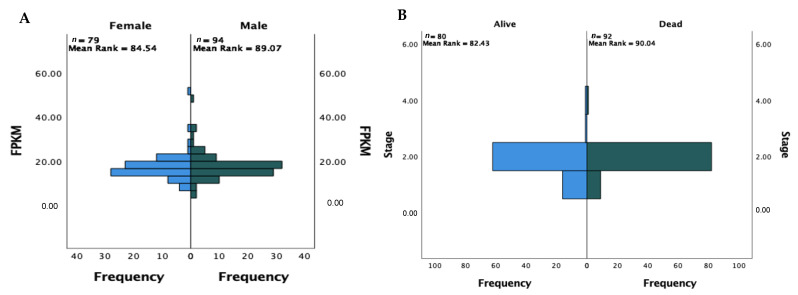
Population pyramids showing the results of an independent samples Mann–Whitney U test of difference on: (**A**) FPKM expression against patient sex in a cohort of 173 pancreatic ductal adenocarcinoma patients. No significant difference was observed between the groups (*U* = 3518.5, *p* = 0.553). (**B**) Patient stage against alive/dead status in a cohort of 172 pancreatic ductal adenocarcinoma patients. No significant difference was observed between the groups (*U* = 4005.5, *p* = 0.119).

**Figure 4:**
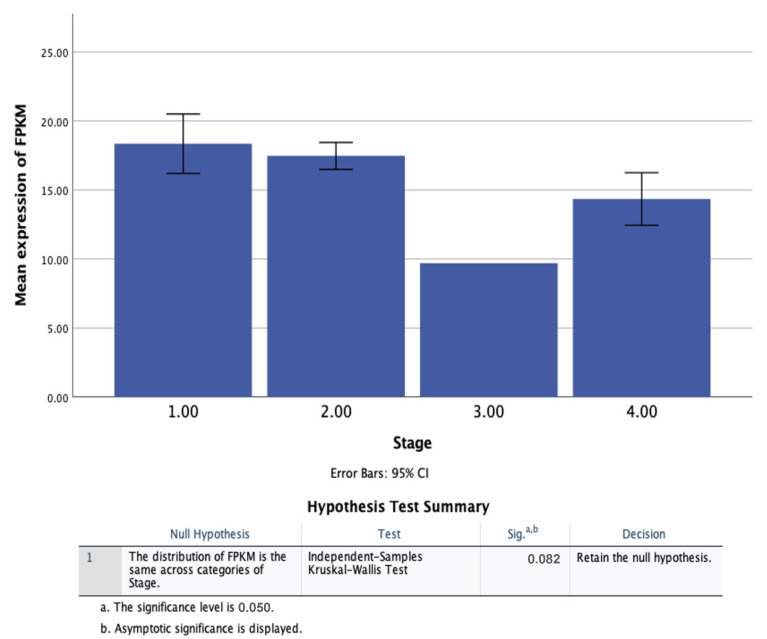
A simple histogram to demonstrate the correlation between expression of FPKM and the cancer stage (1–4) in pancreatic ductal adenocarcinoma patients. There is no correlation between an increase in cancer stage and a change in FPKM expression, as demonstrated by the Kruskal–Wallis test of independence (*p* = 0.082, >0.05).

The mean expression of FPKM in PDAC patients was 17.5202 (±5.81957) and the mean age of patient was 65-years (±11) with a range from 35 to 88 years (Table 2). The following descriptive statistical analysis (Figure 2, Figure 3 and Figure 4) was carried out to preliminary investigate any correlations/relationships between FPKM expression and patient status. 

The distribution of the dataset is visualised with a box plot (Figure 2), showing the median, 25th and 75th percentiles, and outliers (above or below 1.5 times the interquartile range). 

79 females and 94 males were included in this dataset. To understand whether FPKM expression corresponds to the patient’s sex, Mann–Whitney U test was carried out, which showed no significance (*p* = 0.553) between the two groups (Figure 3A). 

The number of patients reported deceased and alive at the time of mRNA sequencing was 92 and 80, respectively. The distribution of patient status across the four cancer stages (i, ii, iii, iv) can be observed in Figure 3B. A similar distribution is observed, and the median score between the patient statuses are not found to be statistically significant, *U* = 4005.500, *p* = 0.119.

#### 3.2.2. Investigating the Correlation between FPKM Expression and Patient Status

Spearman’s rank correlation coefficient (rho) was carried out to investigate the significance of any relationships between FPKM expression and patient supporting information. Table 3 shows that there is no significance between the levels of CDK4 (quantified as FPKM expression) and the patient’s age, time since diagnosis or status (*p* = 0.308; 0.768; 0.114 respectively). However, significance was found between the expression of FPKM and the cancer stage (*p* = 0.031). 

To investigate further the significance found by Spearman’s rho, Table 3, between FPKM expression and the patient’s cancer stage, a simple histogram was presented and Kruskal–Wallis test of independence was performed. The histogram suggests that there is significance between being in cancer stage 3 and the expression of FPKM, however the Kruskal–Wallis analysis shows that this study did not demonstrate any correlation between FPKM expression and cancer stage, *H* (3) = 6.71, *p* = 0.082 (Figure 4). 

**Figure 5:**
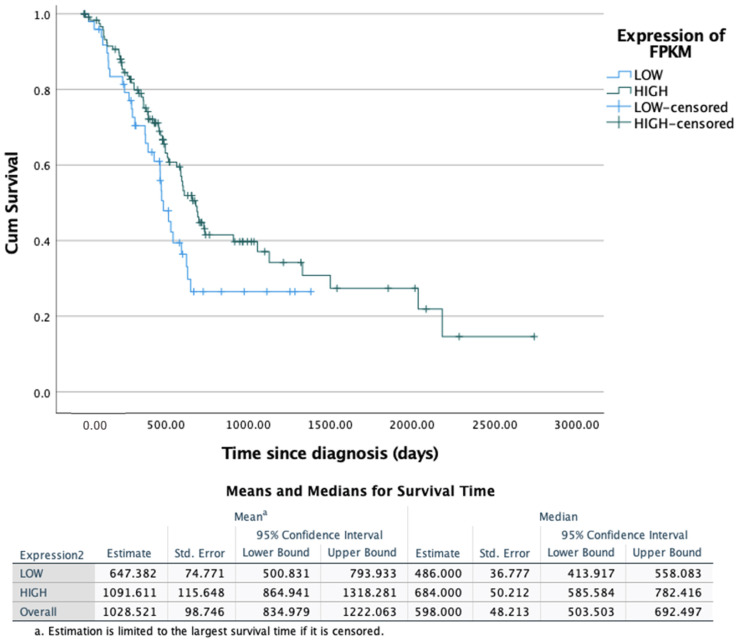
Kaplan-Meier plot showing the survival analysis of CDK4 expression (quantified by expression of FPKM reported) for pancreatic ductal adenocarcinoma patients (*n* = 173). Expression groups determined by: low < 15.07, high > 15.07. No statistical significance was observed (χ^2^(1) = 3.312, *p* = 0.069, >0.05).

Finally, based on the FPKM value for CDK4, patients were classified into two expression groups (high > 15.07, low < 15.06) using the best expression cut-off that yields the maximum difference with respect to the survival of patients from each group and the lowest log rank p-value. The prognosis of each group was then examined by maximally separated Kaplan-Meier survival estimators (Figure 5) to determine if CDK4 has significance on patient survival and is therefore associated with patient survival. The survival distributors for the two expression groups were not statistically significantly different, χ^2^(1) = 3.312, *p* = 0.069. Therefore CDK4 has no prognostic value in PDAC patients. 

### 3.3. Antibody Profiling of CDK4 Protein Expression in PDAC Tissues

**Figure 6:**
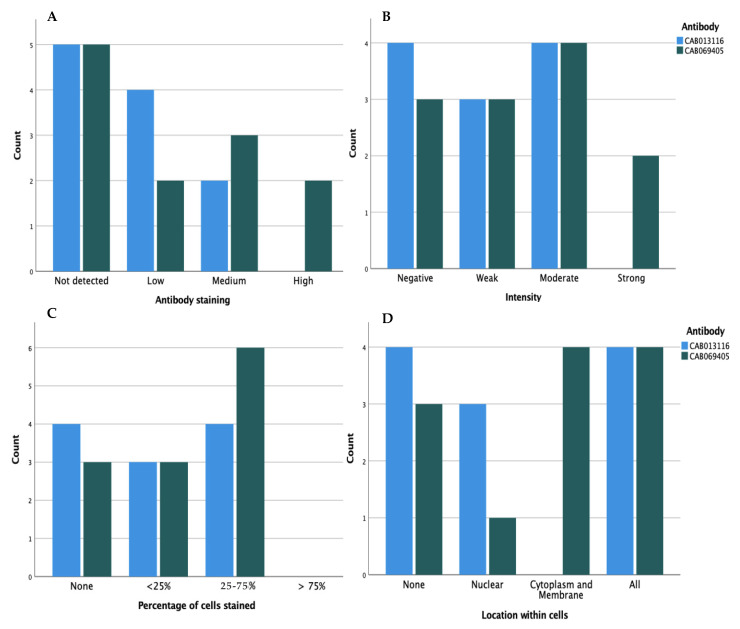
Clustered bar charts showing characteristics of two different CDK4 monoclonal antibodies (1—CAB013116 (ThermoFisher Scientific) and 2—CAB069405 (AbFrontier)) on tissue sections from pancreatic ductal adenocarcinoma patients. (**A**) The antibody staining levels, (**B**) The level of intensity, (**C**) The percentage of cells stained, (**D**) The location within cells.

In total, 11 PDAC histology sections were stained with both CAB013116 and CAB069405, and 1 was stained only with CAB069405. The sections were annotated by trained specialists according to the (i) level of staining (not detected; low; medium; high), (ii) intensity of the stain (negative; weak; moderate; strong), (iii) percentage of cells stained (none, <25%, 25−75%, >75%) and (iv) the location of stain within the cells (none, nuclear, cytoplasm and membrane, all). The results are shown in Figure 6A−D. When staining with both antibodies, CDK4 expression was not detected in almost half of the patients (*n* = 5) (Figure 6A). Of those where CDK4 expression was detected, the expression appears heterogeneous with equal distributions observed across all factors. CAB013116 had a higher number of negative intensity levels than CAB069405, while presented the same levels in the moderate intensity (Figure 6B). No histological sections had staining that occupied more than 75%, with the majority stained across 25−75% (Figure 6C). The majority of staining was found in the cytoplasm, membrane and nucleus for both antibodies (*n* = 4). CAB013116 had a higher number of nuclear stains than CAB069405 (*n* = 3, *n* = 1, respectively) (Figure 6D).

**Figure 7:**
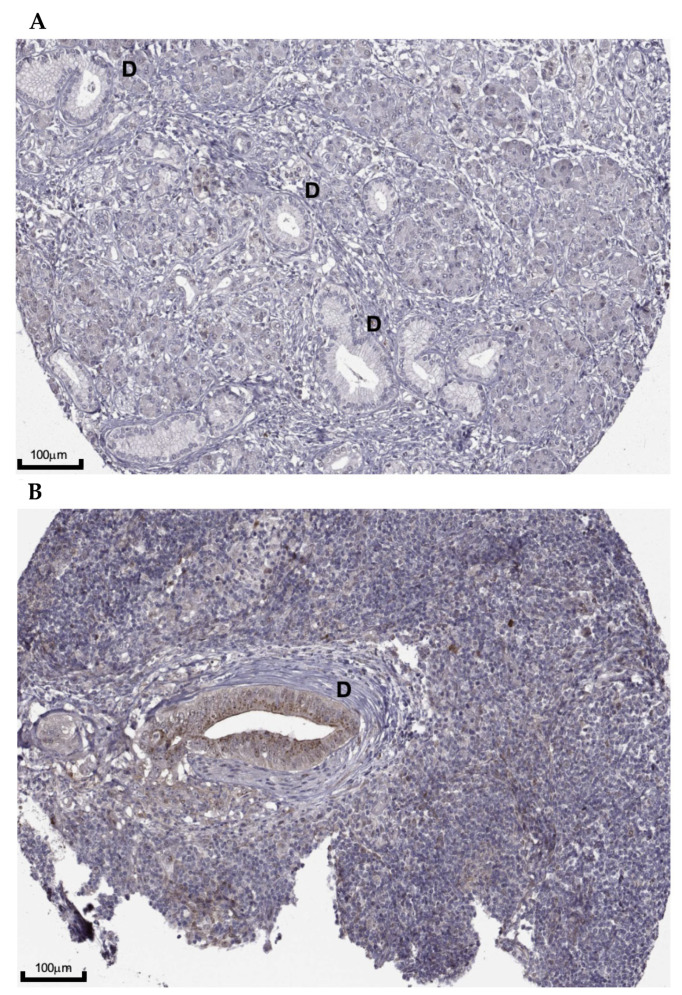
Histological slides from two pancreatic ductal adenocarcinoma patients both stained with a monoclonal antibody for CDK4-CAB069405 (AbFrontier). (**A**) Slide shows no staining detected, with negative intensity, 0% of cells stained and no staining within the cells (scale bar—100mm) (**B**) Slide shows high levels of staining, with strong intensity, 25–75% of cells stained and evidence of staining within the nucleus, cytoplasm and membrane (scale bar—100mm). D = Interlobular duct. Image credit: Human Protein Atlas, image available from vv20.1.proteinatlas.org (accessed on 6 June 2021) [23].

Figure 7 provides an example of the disparity between stains. Figure 7A shows the absence of CDK4 expression, indicated by the lack of brown DAB staining. In contrast, high levels of staining with a strong intensity and occupation of 25−75%, indicated by the abundance of brown staining, are shown in Figure 7B. This difference is highlighted clearly when comparing the interlobular ducts (D) on both stains-CAB069405 was used in both examples. 

### 3.4. KEGG PATHWAY Analysis for CDK4

Table 4 provides a summary of the major pathways and diseases that CDK4 is involved in, as obtained from the KEGG PATHWAY database. Of interest, it appears to be involved in a number of cancers-including pancreatic, bladder, breast, glioma and melanoma- and is important in the regulation of the cell cycle and senescence. 

**Figure 8:**
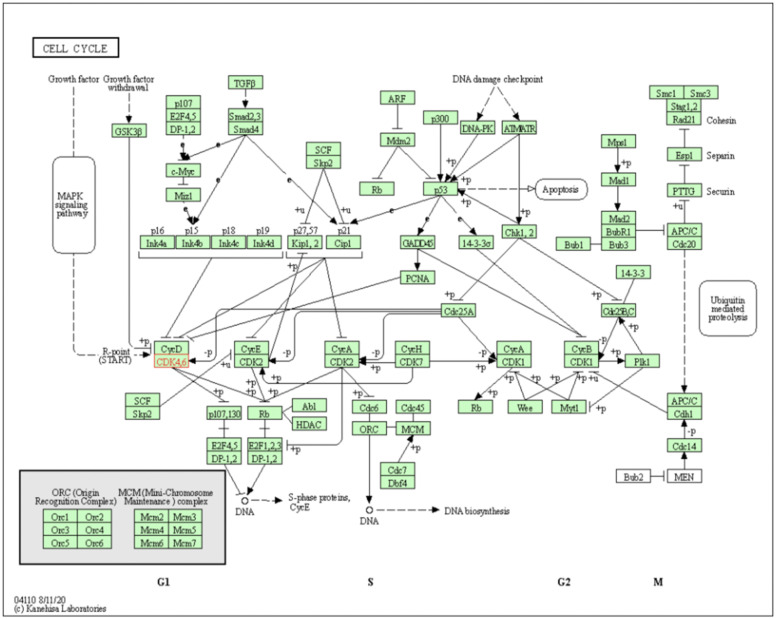
Pathway map showing the mitotic cell cycle progression, through the events of DNA replication (S phase) and mitosis (M phase), separated temporally by the G1 and G2 phases. Cyclin-dependent kinases (CDKs), activated by specific cyclins to form CDK/cyclin-complexes, modulate the activity of key substrates to regulate the cell’s progression through the cell cycle phases. Cyclin-CDK inhibitors (CKIs), including p16^INK4a^, p15^INK4a^, p27^Kip1^ and p21^Cip1^ contribute to the negative regulation of CDK activities. CDK4/6 (the focus of this study) is shown in red. Image taken from: KEGG PATHWAY database (Kyoto, Japan) [25]. Accession number: map04110.

Figure 8 outlines the role that CDK4 (shown in red) plays in the mitotic cell cycle progression. CDK4/6 associates with D-type cyclins to mediate the progression through the G1/S transition. M-phase inducer phosphatase 1 (Cdc25A) activates CDK4/6 through the removal of a phosphate group (dephosphorylation), which leads to hyperphosphorylation of retinoblastoma (RB) tumour suppressor protein and its relation proteins, p107 and p130, resulting in the release of E2F transcription factors. The resulting activation of E2F-mediated transcription, controlled, in part, through cyclin A/CDK2, allows for the cell to transit into S phase and thereby initiating DNA replication. 

**Figure 9:**
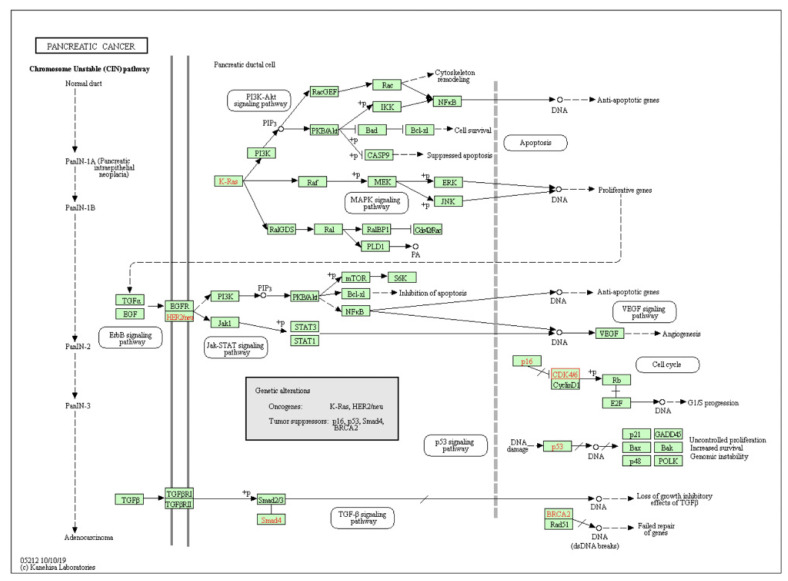
Disease pathway map for pancreatic cancer, where genetic alterations are shown in red. The Chromosome Unstable (CIN) pathway highlights the progression of normal ductal epithelium to infiltrating adenocarcinoma (left) in relation to the accumulation of genetic aberrations (right); the overexpression of HER-2/neu and point mutations in KRAS occur in early events, followed by the inactivation of p16 and the inactivation of p53, SMAD4 and BRCA2 occur in later events. Image taken from: KEGG PATHWAY database (Kyoto, Japan) [25]. Accession number: map05212.

The role of CDK4 in the disease progression of pancreatic cancer summarised in Figure 9. The inactivation of the p16^INK4a^ tumour suppressor gene leads to the activation of CDK4/6-cyclinD1 complex, resulting in the hyperphosphorylation of RB. This then causes the release of E2F transcription factors allowing for the cell to progress through the G1/S transition. The loss of p16^INK4a^, and therefore the loss of CDK4/6 inhibition, leads to uncontrolled cell proliferation. 

## 4. Discussion

PC is a devastating and rapidly progressive malignancy that continues to have a very poor 5-year survival rate of 8%. Despite major advances in the understanding of the pathogenesis of PC, owing to the development and large scale use of next-generation sequencing (NGS), the diagnosis and therapy of PC remains a formidable challenge. Due to the aggressiveness of the disease and the lack of effective early stage symptoms and screening methods, more than half of PC patients are diagnosed at advanced stages and are ineligible for resection-the only curative treatment available [26]. There is a clear unmet need for novel interventions that provide favourable patient survival outcomes. 

### 4.1. Pancreatic Cancer Epidemiology

An estimated 495,773 new cases and 466,003 deaths were reported globally in 2020 (Table 1), and both incidence and mortality rates are shown to be higher in males than females, with a ratio of 1.13:1. This pattern is consistent across other worldwide epidemiological studies, though the reason, and aetiology, remains insufficiently known [27]. One suggested explanation is the increased exposure to environmental or occupational risk factors, such as heavy smoking or drinking. Cigarette smoking has shown to be five times greater in males (24%) than females (5.4%) [28] and is a well-established environmental risk factor of PC, approximately 25% of PC cases attributed to cigarette smoking [29]. Specifically, smoking induces DNA methylation and creates DNA adducts—a length of DNA covalently bonded to a chemical, such as one of the three dozen tumorigenic chemical species contained in a cigarette [30]—which collectively aggregate and have been associated with tumorigenic mutations, including the activation of *KRAS* [31]. The risk of PC has been shown to decrease rapidly following a few years of cessation, though it may take up to 20 years for that risk to reach the same level as those who have never smoked [32]. Despite this, the disparities in incidence rates between males and females remains unclear and could also be down to an undiscovered genetic factor. 

When comparing the ASR per 100,000, the incidence rates were highest in Northern America (ASR, 8), Europe (ASR, 7.8) and Oceania (ASR, 6.6) and the lowest in Africa (ASR, 2.3). While a clear explanation is lacking for these observed differences between countries, variations in the levels of obesity, smoking, physical inactivity and diabetes could be a leading cause [33]. Additionally, the quality of cancer registries and the availability of accurate diagnostic techniques can lead to discrepancies in the number of reported cases between countries and varying levels of coverage and completeness. Furthermore, when comparing global incidence rates based on HDI levels, the highest rates tended to predominate in regions with very high or high HDIs, and the lowest rates were observed in regions with medium and low HDIs (Figure 1A). This could be attributed to lifestyle factors associated with westernisation and recognised risk factors of PC, such as obesity and smoking, or the increased awareness of health/prevention schemes and availability of imaging techniques seen in regions of higher HDIs [27]. 

Similar trends are seen when comparing the mortality rates of PC, with the highest numbers equally observed in Europe and Northern America and countries with very high and high HDIs (Figure 1B), and the lowest numbers seen in Africa and countries with medium to low HDI. Both incidence and mortality rates have continued to rise over the past two decades (Figure 1C,D), much of which is caused by the population aging, where age is the strongest risk factor of PC, with the median age at diagnosis being 71 years and only 20% of cases developing before 60 years, and 3% prior to the age of 45 [34]. 

Nonetheless, these estimates are unable to reflect the impact of the coronavirus disease 2019 (COVID-19) caused by the severe acute respiratory syndrome coronavirus 2 (SARS-CoV-2), as they are based on extrapolations of cancer data collected prior to the pandemic. While the full extent of the impact of the COVID-19 pandemic, especially in and between world regions, remains uncertain, the closure of health systems and suspensions to screening programs has undoubtedly caused delays in diagnosis, treatment and availability to care. As such, there is expected to be a short-term decline in reported PC incidences followed by an increase in advanced-stage diagnoses and fatalities [35]. 

### 4.2. CDK4 Expression in Pancreatic Cancer Patients

CDK4, a serine/threonine kinase that modulates cell cycle entry through the phosphorylation of RB, is frequently activated in human cancers and has become a common target of interest for new therapeutics. The CDK4/6 inhibitors (CDK4/6i) Palbociclib, Ribociclib, and Abemaciclib have recently gained FDA-approval and shown success in the treatment of advanced metastatic hormone receptor-positive (HR+)/HER2-negative breast cancer [15], with several clinical trials now testing their effects in multiple tumour types. However, the therapeutic efficacy of monotherapy CDK4/6i in PC has shown to be largely undermined by compensatory cell cycle regulatory pathways, including the upregulation of Cyclin E1 [36] and the loss of RB [37]. Preclinical data has also suggested that the inhibition of CDK4/6 antagonises the effect of several types of chemotherapy, such as in combination with gemcitabine (the most common chemotherapy agent used in advanced PC [38]), by blocking chemotherapy-induced cytotoxicity. Although, when used with a large panel of MEK and P13K/mTOR inhibitors, high reproducible additive effects were observed [36]. This combination has also shown success in *KRAS* mutant colorectal cancer [39]. Still, the exact clinical potential and use of CDK4/6i in PC requires further exploration and remains undecided. 

To provide an overview, and gain an understanding of the function and role that CDK4 plays in PC patients—which could provide useful when assessing the efficacy of successive CDK4/6i for the treatment of PC- RNA-Seq data from the TCGA was analysed with the use of FPKMs to quantify CDK4 gene expression. For 173 PDAC patients, the mean expression of FPKM was 17.5202 (±5.8195) with a range of 5.80 to 52.10, and expressed in all patients (Figure 2). The mean age of the patient was 65 years (±11), similar to the average age of PC patients in a number of epidemiological studies, and the mean time since diagnosis was 557 days (Table 2). No significant differences were found between the FPKM levels and sex (*U* = 3518.5, *p* = 0.553) (Figure 3A), age (*p* = 0.308), time since diagnosis (*p* = 0.768) or patient status (*p* = 0.114) (Table 3). As a result, there appears to be little evidence to suggest the need to explore specific target groups that could be responsible for different responses to treatment based on these clinical factors alone in the investigation of new CDK4/6i. 

However, preliminary significance was found between the expression of FPKM and the patient’s cancer stage when using Spearman’s rank correlation coefficient (*p* = 0.031), though when investigated further no difference was observed between the stage groups (overlapping error bars and *p* = 0.082 (Kruskal–Wallis test) in Figure 4). This discrepancy is likely attributed to the limited number sample sizes obtained from the HPA, where very few (*n* = 1 and *n* = 2, respectively) patients reported are in stages 3 and 4 (Supplementary Table S1). Further studies that aim to reduce this discrepency and have more equal and representative numbers across all stages could provide more insight. CDK4 positivity has been correlated with a high tumour grade in breast cancer, though similarly no associations between any other clinical factors were found [40]. Survival analysis (Figure 5) also demonstrated that CDK4 is not a prognostic gene for PC, hence offering little evidence as a potential biomarker.

While no overall significant differences were observed between any of the aforementioned clinical factors (despite CDK4 expression seen in all patients), a larger population size (greater than 173), which also evaluated the risk in other subtypes of PC, including PanNETs, would establish a more thorough understanding into the expression of CDK4 in PC patients. Further studies should also evaluate the expression of CDK4 in normal pancreatic tissue samples for comparison, using the same normalisation (FPKM) methods and experimental platforms, as this crucial information was missing from the HPA/TCGA. 

The protein expression of CDK4 was also evaluated by immunohistochemistry, with levels expressed in only half of patients (Figure 6A). This is dissimilar to the findings observed for the mRNA expression in patients (seen in all) and could be a result of low levels of CDK4 gene transcription in PC or limitations by the stage of cell cycle at the time the samples were tested. Differential expressions between the PDAC patients were also observed, with varying intensities, levels of cells stained and location within the cell (Figure 6B–D). The absence of clinical information provided for these samples from the HPA thereby lacks the prognostic value of mRNA expression and protein abundance, and larger studies are required to assess the relationship between CDK4 mRNA levels, the corresponding protein expression and the association with patient prognosis or responsiveness to CDK4/6i. In addition, since the activation of CDK4 in PC is due to either the upregulation of cyclin D1 or the loss of p16^INK4a^ (Figure 9), subgrouping patients based on cyclin D and p16^INK4a^ levels could show varying and important differences in response levels to CDK4/6i. Also required is an indication of the varying expression levels between the types of PC, such as exocrine vs endocrine tumours. All of these results combined could offer critical insights and direction into future targeted treatments, as proposed by Kosti et al. (2016) [41], who hypothesised that testing a patient for highly correlated mRNA transcript before the administration of treatment with a drug against the corresponding protein (e.g., levels of CDK4 mRNA transcript levels before the use of CDK4/6i) could improve treatment target specificity and therefore treatment outcomes. 

### 4.3. The Role of CDK4 and the Current Use of CDK4/6 Inhibitors in Pancreatic Cancer

Figure 9 outlines the basic pathogenic role of CDK4 in PC, where the inactivation of the p16^INK4a^—an endogenous inhibitor of CDK4/6 encoded by *CDK2NA*—activates the CDK4/6-cyclinD1 complex resulting in the hyperphosphorylation of RB, release of E2F transcription factors and uncontrolled cell proliferation. The activity of CDK4/6 is also known to be upregulated by the overexpression of cyclin D1, observed in 25% of PC cases [42]. *Six1* transcription factor gene, known to be overexpressed and induce epithelial-mesenchymal transition (EMT) in PC [43], has been shown to upregulate the expression of cyclin D1 [44]. Several signalling pathways, including Wnt/β-catenin, MAPKs and P13K/AKT/mTOR similarly upregulate cyclin D1 expression, inducing the association to CDK4 [45]. Notably, mTOR is stimulated independently of the P13K signalling pathway by CDK4, through the phosphorylation of tumour suppressor folliculin (FLCN) and promotion of lysosomal function, leading to increased cancer cell survival [46]. Interestingly, the combination of mTOR inhibitors has been shown to enhance response to CDK4/6i in PDAC [47]. Another promising combination is the potential use of locally administered microRNA-206 (which has been shown to target the RTK-Ras-MAPK-P13K/Akt-CDK pathway) and CDK4/6i, such as Abemaciclib, following successful in vitro and in vivo studies in malignant pleural mesothelioma [48]. 

Conventional PDAC treatments are based on chemotherapies, including taxanes, and DNA-damaging agents (gemcitabine). CDK4 inhibitors interfere with these therapeutics, by arresting the G_1_ phase and disrupting their mechanisms of action in later stages of the cell cycle. However, sequential administration of CDK4/6i after taxane treatment further prevents cellular proliferation by the repression of homologous recombination DNA repair. CDK4/6i could be administered before/alongside S-phase or mitotic-targeting compounds; in other cancers (including ovarian and bladder), this form of administration can lead to protection from the cytostatic or cytotoxic effects of CDK4/6i [49]. 

Interestingly, those patients with high expression of RB protein might respond more favourably to this form of combination treatment. RB expression seems to guide the potent CDK4/6i PD-0332991 (palbociclib), which improves chemosensitivity and disrupts the extracellular matrix (ECM), when used in combination with conventional chemotherapeutics [50]. 

While this study aimed to evaluate the role that CDK4 itself plays in the pathogenesis of PC, it is clear that further studies should also investigate the expressions and impact of CDK6 and the CDK4/6-cyclin D1 complex. 

## 5. Conclusions

As mentioned, the critical role of CDK4 in the pathogenesis of PC, and the recent success of CDK4/6i in breast cancer, makes CDK4-inhibition a promising target for future therapeutics in the treatment of PC. However, studies have demonstrated that the degree of cell cycle inhibition of CDK4/6i in PDAC is significantly more modest compared to the response observed in breast cancer models. Therefore, efforts have focused on combinatorial approaches, such as the use of MEK and mTOR inhibitors [51]. Also, the combination of CDK4/6i with traditional chemotherapies have been reported to have both synergistic and antagonistic cellular responses in PC. The full extent of CDK4/6i and use in PC is yet to be achieved and the current underlying basis for optimal combinatorial activity remains undetermined. 

## Figures and Tables

**Table 1:** Estimated pancreatic cancer incidence and mortality rates by continent and sex in 2020. Adapted from: GLOBOCAN, 2020 [17] * ASR-Age-standardised rate per 100,000 ** Cumulative Risk per 100.

**Population**		**Estimated Incidence**						**Estimated Mortality**				
	**Male**			**Female**			**Male**			**Female**		
	***n***	ASR *	Cum. Risk **	***n***	ASR *	Cum. Risk **	***n***	ASR *	Cum. Risk **	***n***	ASR *	Cum. Risk **
Asia	129,488	4.7	1.5	104,213	3.3	1.27	123,337	4.5	1.5	100,697	3.1	1.27
Europe	70,210	9.4	2.67	6906	6.4	2.04	66,698	8.8	2.59	65,436	5.8	1.98
Northern America	32,938	9.3	2.7	29,705	6.9	2.1	27,888	7.6	2.38	25,389	5.5	1.87
Latin America and the Caribbean	18,477	5	1.55	18,875	4	1.36	17,897	4.9	1.53	18,133	3.8	1.33
Africa	9239	2.7	0.79	7831	2	0.58	8936	2.6	0.78	7613	1.9	0.58
Oceania	2513	7.3	2.37	2378	6	2.03	2084	6	2.04	1895	4.5	1.7
**Population**		**Total**						**Total**				
	***n***		ASR *		Cum. Risk **		***n***		ASR *		Cum. Risk **	
Asia	233,701		4		1.38		224,034		3.8		1.38	
Europe	140,116		7.8		2.31		132,134		7.2		2.21	
Northern America	62,643		8		2.37		53,277		6.5		2.1	
Latin America and the Caribbean	37,352		4.5		1.45		36,030		4.3		1.42	
Africa	17,070		2.3		0.67		165,349		2.3		0.66	
Oceania	4891		6.6		2.19		3979		5.2		1.86	
	495,773						466,003					

**Table 2:** Descriptive statistics of the data collected from The Cancer Genome Atlas (TCGA) for 173 pancreatic ductal adenocarcinoma patients, in relation to their age (years), time since diagnosis (days) and expression of CDK4 (quantified as expression levels of FPKM) [23].

Patient Status	*n*	Minimum	Maximum	Mean	Std. Deviation
Age (years)	173	35.00	88.00	64.7283	±10.87033
Expression of FPKM	173	5.80	52.10	17.5202	±5.81957
Time since diagnosis (days)	173	4.00	2741.00	556.7052	±461.29197

**Table 3:** Correlation between the expression of FPKM and patient’s age, stage, time since diagnosis and status for 173 pancreatic ductal adenocarcinoma patients, using Spearman’s rank correlation coefficient. Significance was observed between FPKM expression and cancer stage. * value is statistically significant.

Analysis		Age (Years)	Stage	Time Since Diagnosis (Days)	Alive or Dead Status
FPKM	Correlation Coefficient	−0.078	−0.164 *	−0.023	−0.121
	Sig. (2-tailed)	0.308	0.031 *	0.768	0.114
	*n*	173	173	173	172

**Table 4:** A list of biological pathways and diseases that CDK4 is involved in, as obtained from the Kyoto Encylopedia of Genes and Genomes (KEGG) PATHWAY database (Kyoto, Japan).

CDK4	Cyclin-Dependent Kinase 4
Pathways	(1) Endocrine resistance, (2) Cell Cycle, (3) p53 signalling pathway, (4) P13K-Akt signalling pathway, (5) Cellular senescence, (6) Tight junction, (7) T-cell receptor signalling pathway. (8) AGE-RAGE signalling pathway in diabetic complications, (9) Cushing syndrome, (10) Hepatitis C, (11) Measles, (12) Human cytomegalovirus infection, (13) Influenza A, (14) Human papillomavirus infection, (15) Human T-cell leukaemia virus 1 infection (15) Kaposi sarcoma-associated herpesvirus infection, (16) Epstein-Barr virus infection, (17) Pathways in cancer, (18) Viral carcinogenesis, (19) Pancreatic cancer, (20) Glioma, (21) Melanoma, (22) Bladder cancer, (23) Chronic myeloid leukaemia, (24) Small cell lung cancer, (25) Non-small cell lung cancer, (26) Breast Cancer, (27) Hepatocellular carcinoma
Disease	(1) Glioma (amplification) (2) Melanoma (mutation) (3) Cervical cancer (overexpression)

## Data Availability

A number of publicly available datasets were analysed in this study. This data can be found at: (1) https://gco.iarc.fr/today/home; (2) http://ci5.iarc.fr; (3) https://www.proteinatlas.org/ENSG00000135446-CDK4/pathology/pancreatic+cancer; (4) Kyoto Encyclopedia of genes and genomes at doi:10.1093/nar/28.1.27, reference number [25].

## Supplementary Materials

The following are available online at https://www.mdpi.com/article/10.3390/healthcare9111478/s1, Table S1: The number of pancreatic ductal adenocarcinoma patients (*n* = 173) in each cancer stage (1–4) at the time of the study collected from The Cancer Genome Atlas (TCGA). Figure S1: The annual percent changes for the incidence rates of pancreatic cancer patients for the years 1995 to 2011, Figure S2: The annual percent changes for the mortality rates of pancreatic cancer patients for the years 1995 to 2011.
