# Supplementary material for: The Role of CDK4 in the Pathogenesis of Pancreatic Cancer
Source: Healthcare (Basel). 2021 Oct 30;9(11):1478. doi: 10.3390/healthcare9111478 (PMC8620733; doi:10.3390/healthcare9111478)
Supplement: Supplementary file 1 [file healthcare-09-01478-s001.zip › healthcare-1368574-supplementary.pdf]

Supplementary Materials

Table S1. The number of pancreatic ductal adenocarcinoma patients (*n* = 173) in each stage (1 – 4) at the time of the study collected from TCGA.

Stage	Frequency	Percent (%)	Cumulative Percent (%)
1	25.0	14.5	14.5
2	145	83.8	98.3
3	1.00	0.60	98.8
4	2.00	1.20	100
	173	100	

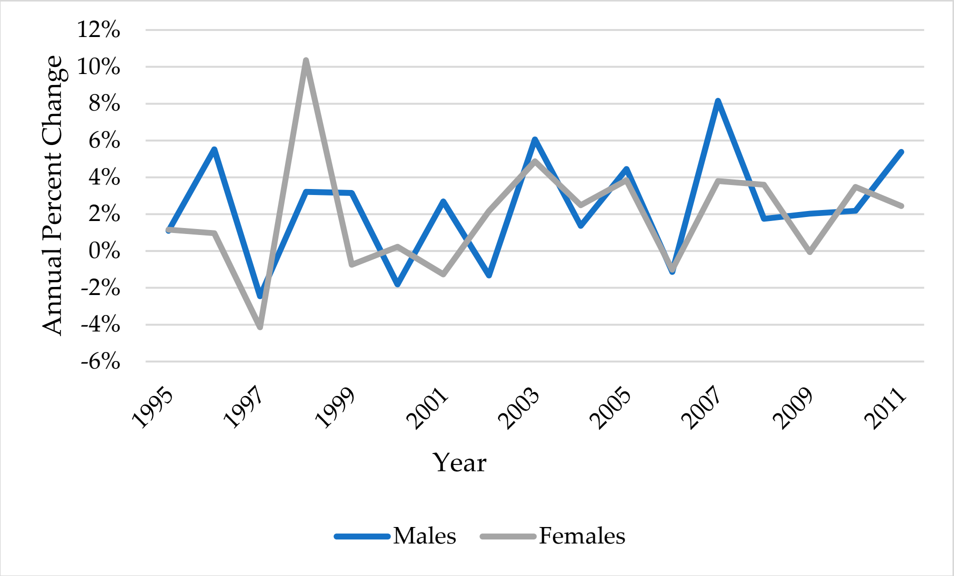

Figure S1. The annual percent changes for the incidence rates of pancreatic cancer patients for the years 1995 to 2011.

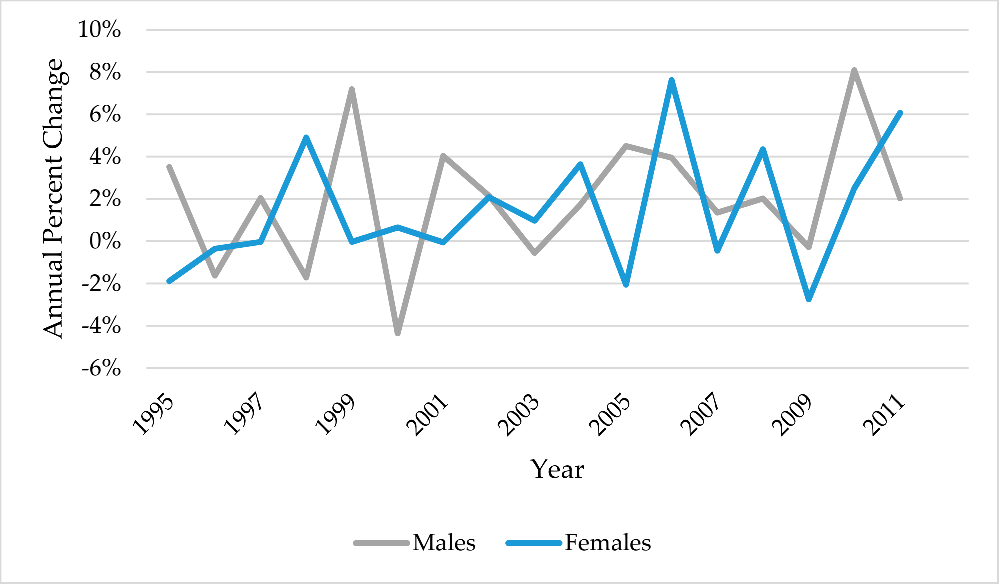

Figure S2. The annual percent changes for the mortality rates of pancreatic cancer patients for the years 1995 to 2011.
